# Potential of Homopolysaccharide-Producing Starter Cultures in the Fermentation of Coconut Yoghurt Alternatives Enriched with Pea Protein Isolate

**DOI:** 10.3390/foods15010048

**Published:** 2025-12-23

**Authors:** Sophie Libberecht, Mia Ristevska, Monika Gibis, Myriam Loeffler

**Affiliations:** 1 Meat Technology & Science of Protein-Rich Foods (MTSP), Department of Microbial and Molecular Systems, Leuven Food Science and Nutrition Research Centre, KU Leuven Campus Ghent, 9000 Ghent, Belgium; 2 Institut Agro, Master 2 Microbiology and Physicochemistry of Food and Wine Processes, 21000 Dijon, France; 3 Department of Food Material Science, University of Hohenheim, 70593 Stuttgart, Germany; monika.gibis@uni-hohenheim.de

**Keywords:** fermentation, microbial in situ homopolysaccharide formation, protein-fortification, plant-based yoghurt analogues, texture

## Abstract

This study investigates the use of a homopolysaccharide (HoPS)-producing *Latilactobacillus sakei* strain for the production of protein-enriched plant-based yoghurt analogues based on coconut milk. Formulations varied in added sucrose (2.5% or 5.0% *w*/*w*), pea protein isolate (PPI; 0–5.0% *w*/*w*), and tapioca starch (0%, 1.5% *w*/*w*), and were fermented with either a HoPS-producing strain (*L. sakei* 1.411), or a non-exopolysaccharide (EPS)-producing control strain (*L. sakei* 1.2037) with very similar acidification kinetics. Microbial growth and pH were monitored, HoPS content was determined via HPLC, and both firmness and syneresis were assessed during 5 days of storage at 4 °C. EPS yields were significantly higher (*p* < 0.05) in samples with 5.0% *w*/*w* added sucrose compared to those with 2.5% *w*/*w*. Fermentation with *L. sakei* 1.411 generally resulted in firmer gels (*p* < 0.05) and reduced syneresis (*p* < 0.05) compared to *L. sakei* 1.2307 and the enhanced viscosity (sample thickness) was also observed in a sensory analysis. Samples containing starch and 5.0% *w*/*w* PPI showed the highest firmness-related values. These findings demonstrate the potential of in situ HoPS production to improve the texture and stability of protein-enriched coconut-based yoghurt analogues. It highlights the importance of matrix formulation, strain selection and process control, which all contribute to the final product quality.

## 1. Introduction

The transition toward a more sustainable food system represents a global challenge. Efforts are increasingly directed at reducing environmental impacts through improved water and land use, lowering greenhouse gas emissions, and promoting dietary shifts from animal-based to more plant-based foods [1]. This shift is evident in the growing market for plant-based products, including plant-based dairy alternatives with an estimated rise in the global compound growth rate by 9% by 2032 [2], and the expanding range of vegan product alternatives available in retail. Public health authorities and dietary guidelines are also supporting this transition, recommending a rebalancing of protein intake from an animal-to-plant protein intake ratio of 60:40 to a 40:60 contribution, which is also in alignment with nutritional goals [3]. Fermented dairy products such as yoghurts are widely consumed food products that are associated with various health benefits [4]. Accordingly, there is a growing interest in developing plant-based yoghurt alternatives that do not only align with consumer expectations regarding appearance, texture, mouthfeel and taste but also address increasing demands for clean(er) labels, minimal processing, and improved nutritional profiles [5,6,7]. Since the type and hence, the techno-functional properties of proteins in dairy and plant-based matrices differ remarkably, the development of yoghurt analogues is associated with a number of challenges.

In dairy yoghurt manufacture, bovine milk is usually standardized and heat-treated prior to fermentation, leading to the denaturation of β-lactoglobulin, which can subsequently interact with caseins to improve yoghurt gel structure. After homogenization and cooling to around 42–45 °C, the milk is inoculated with starter cultures (usually *Streptococcus thermophilus* and *Lactobacillus delbrueckii* subsp. *bulgaricus*) that ferment the carbon source lactose into lactic acid [8]. This acidification process causes the pH to decrease, leading to the acid-induced aggregation/coagulation of casein micelles (IEP pH ~4.6) and to the formation of a gel network that is further stabilized through the aforementioned interaction of whey proteins with κ-casein on the micelle surface [9]. While the fundamental principles of yoghurt manufacture are well-established, recent studies have focused on developing advanced approaches to further optimize textural stability and enhance the sensory profile of plain yoghurt products. For instance, Fan, et al. [10] addressed this through co-fermentation using binary probiotic cultures (*Lacticaseibacillus casei* CGMCC1.5956 and *Levilactobacillus brevis* CGMCC1.5954) combined with a water-soluble plant extract from *Semen Ziziphi Spinosae* [10] or in conjunction with wolfberry dietary fibre [11], both leading to yoghurt products with improved gel characteristics and high odour scores. The functionality of plant proteins differs remarkably from that of dairy proteins and is strongly influenced by their processing history. Klost and Drusch et al. [12,13], who investigated the gelling properties of pea proteins, reported that fermentation induces self-supporting gels through a two-step mechanism involving the formation of a percolated network, followed by the condensation of smaller aggregates. Protein–protein interactions were found to be primarily hydrophobic, dominated by the legumin fraction, with minor contributions from electrostatic interactions between vicilin and basic legumin-β chains, leading to the partial incorporation of vicilin into the gel [12]. The differences in gelation properties between plant-based and animal-based proteins are the subject of ongoing research, and the key factors influencing gelation have been comprehensively discussed in recent reviews [14,15]. When applied as concentrates or isolates, plant proteins often undergo partial to (almost) full denaturation, which alters their structural integrity and solubility [16]. These changes affect their functional properties in food matrices, including, i.e., the gelling and/or emulsifying properties [17], which in turn impact structure—and hence, the texture of food products [18]. To address these challenges in plant-based dairy alternatives, manufacturers often use hydrocolloids such as gums, starches, or pectin to enhance the stability and organoleptic properties of the final products [19]; however, they need to be declared on product labels. The food industry is hence seeking solutions that tackle both the aforementioned organoleptic challenges and the growing consumer demand for cleaner(er) ingredient lists while also putting more emphasis on the nutritional quality aspects while guaranteeing product safety.

One promising solution could be the use of fermentation with exopolysaccharide (EPS)-producing lactic acid bacteria (LAB), as the in situ formed EPS have been shown to contribute to structure and texture formation, improve rheological properties and to enhance water-holding capacity in dairy products, including yoghurts and yoghurt-based drinks [8,20,21,22]. EPS can be categorized into two distinct classes: namely, homopolysaccharides (HoPS) and heteropolysaccharides (HePS). The former is constituted of a single monosaccharide type (mainly glucose or fructose), whereas the latter is comprised of two or more different monosaccharide units. The synthesis of HoPS is a relatively low energy demanding process, while their molecular weight (>10^6^ Da) and yield (up to a few g/L) are notably high. Conversely, HePS demand a more energy-consuming and complex production pathway, with their molecular weight (10^4^ to 10^6^ Da) and yield (mg/L range) usually being lower [23]. The type and yield of EPS produced, however, depends on several factors, including medium composition and available carbon source, the bacterial strain-, as well as the growth conditions (temperature, pH, incubation time, etc.) used, with stress conditions often promoting EPS production [24]. EPS are produced to facilitate protection against unfavourable environmental conditions and to promote cell-to-cell communication, as reviewed by other authors [24,25]. The current literature indicates that a limited number of LAB strains are frequently used in fermentation processes for plant-based dairy yoghurt alternatives; namely, those usually applied in dairy yoghurt production [26,27,28]. Given that vegan analogues rely on plant materials and do not contain lactose as a carbohydrate source, it is also very interesting to explore the potential of (other) HoPS-producing LAB strains in those matrices as well. This is particularly relevant for plant-based matrices such as protein-enriched coconut yoghurt, which differs substantially in composition from traditional dairy yoghurt. Our study highlights the potential of a mesophilic LAB strain *Latilactobacillus sakei* 1.411 in yoghurt-alternative manufacture, a strain that has been isolated from sauerkraut. This strain has been successfully used for HoPS production in very complex food matrices containing high protein and fat contents. Notably, *L. sakei* 1.411 was capable of producing significant amounts of dextran [29] in spreadable raw fermented sausage products, enabling partial fat replacement by 50% in German Teewurst [30] and allowing for pH modulation closer to the isoelectric point (IEP) of meat proteins [31], while maintaining product spreadability and without negatively affecting sensory perception. In addition to addressing texture-related challenges, fermentation can also contribute to the nutritional profile and microbiological safety of plant-based alternatives. Plant proteins are commonly associated with antinutritional factors such as saponins, tannins, phytic acid, trypsin inhibitors and α-galactosides, which can impair protein digestibility and vitamin or mineral bioavailability [32]. Fermentation has been shown to reduce the levels of these compounds, thereby enhancing the nutritional quality of plant-based alternative products [33]. Moreover, some LAB have the ability to produce B-vitamins and the formed EPS themselves may have prebiotic or immunomodulatory effects [34,35]. In addition, fermentation has been successfully applied to mitigate “off flavors” and “undesirable” aroma compounds often associated with plant proteins [36], which is particularly relevant in the context of protein fortification in plant-based dairy alternatives. For instance, yoghurt alternatives derived from cereals or nuts typically contain low protein contents (often less than 1%) [37]. Fortification with other plant proteins can improve their nutritional value [38] but may also influence structural, and hence textural properties. Among these, pea protein has gained increasing attention due to its affordability, availability, and good digestibility as well as lower allergenic potential compared to soy proteins [39]. Pea protein isolate (PPI) has also been shown to promote gel network formation in plant-based yoghurt analogues following heat treatment and fermentation [40]. However, the utilization of protein isolates in dairy analogue production also raises concerns regarding product safety, as plant protein powders often contain bacterial spores that can germinate under favourable processes or storage conditions. Although plant-based dairy alternatives typically undergo a heat treatment step, heat-resistant spores, such as those of *Bacillus cereus*, can remain in the matrix. Fermentation with (fast-acidifying) LAB to pH values ≤ 4.5, can help suppress the germination and outgrowth of such spores, thereby contributing to the microbiological safety of the final product [41,42,43]. In addition, certain LAB strains are known to produce bacteriocins during fermentation, which can further inhibit the growth of spoilage organisms and pathogens, providing an additional layer of protection [44]. As with dairy yoghurt, fermentation should hence be regarded as the essential processing approach for plant-based yoghurt alternatives. However, achieving a balanced interplay between acidification, gel network formation, and EPS production remains challenging.

A comprehensive understanding of the interrelationships among these factors is essential for advancing future product development, which we aim to contribute to with the present study. Unlike most research on plant-based yoghurt analogues that relies on traditional dairy starter cultures, this work investigates the use of a mesophilic HoPS-producing *Latilactobacillus sakei* strain, previously recognized for its strong performance in complex meat matrices, to improve the texture and stability of pea protein-enriched coconut yoghurt. This approach addresses a critical gap in adapting non-dairy matrices for fermentation by leveraging in situ HoPS production as a natural structuring mechanism. Specifically, this study examines the potential of the HoPS-producing LAB *L. sakei* 1.411 in the formulation of a plant-based yoghurt analogue using coconut milk as the base. This matrix was then supplemented with sucrose (2.5% and 5.0% *w*/*w*) and enriched with tapioca starch (0% and 1.5% *w*/*w*) and/or PPI (0%, 2.5% and 5.0% *w*/*w*). The effects of these compositional variables on gel formation, HoPS production and the resulting product properties were analyzed and compared to products fermented with a non-EPS producing strain exhibiting very similar acidification kinetics.

## 2. Materials and Methods

### 2.1. Raw Material and Chemicals

Ultra-high temperature (UHT) coconut milk (Aroi-D, Bangkok, Thailand), and table sugar (Everyday, Belgium) were obtained from a local supermarket in Ghent, Belgium. Based on the product label, 100 mL of coconut milk contains 19 g fat, 2.0 g carbohydrates, 1.6 g protein, and 0.05 g salt. Tapioca starch was provided by Cargill (San van Gent, The Netherlands), whereas the pea protein isolate (Pisane^®^ M9; protein content 86% on dry matter basis) was supplied by Cosucra Group (Warcoing, Belgium). The starter cultures, *Latilactobacillus sakei* TMW 1.2037 and *Latilactobacillus sakei* TMW 1.411, were obtained from TUM School of Life Sciences (Munich, Germany; LAB characterized in the joint project: AiF 18357 N) and are available in the in-house culture collection. De Man, Rogosa and Sharpe (MRS) agar and broth, and Plate Count Agar (PCA) were sourced from VWR (PA, USA) and peptone was obtained from Carl Roth GmbH & Co. KG (Karlsruhe, Germany). Trichloroacetic acid (99.8%) was supplied by VWR (Randor, PA, USA), and the ion exchange resins Dowex 50WX4 hydrogen form (cation) and Dowex 66 free base (anion) were purchased from Supelco (Bellefonte, PA, USA). Perchloric acid (70%) was obtained from Sigma-Aldrich Chemie GmbH (Munich, Germany) and ethanol (99.8%, analytical reagent grade) was purchased from Fischer Scientific^TM^ (Walthem, MA, USA).

### 2.2. Starter Culture Reactivation

Stock cultures of the bacterial strains *L. sakei* 1.2037 (non-EPS-producing control strain) and *L. sakei* 1.411 (HoPS-producing strain) were prepared in MRS broth (incubated for 24 h at 30 °C) and maintained at -80 °C in 50% (*v*/*v*) glycerol. Prior to experimental use, the cultures were reactivated on MRS agar plates to assess viability. Afterwards, strains were grown in MRS broth prior to conducting initial characterization experiments. For the production of the yoghurt analogues, cultures were also pre-grown in coconut milk supplemented with sucrose (as subsequently described in Section 2.4.1).

### 2.3. Starter Culture Characterization

Initial characterization of the two strains focused on their capacity to produce HoPS under varying incubation temperatures. For this purpose, the reactivated strains were cultured on MRS agar plates supplemented with 2.5% (*w*/*v*) sucrose, allowing visual qualitative assessment of sucrose-dependent HoPS production. The incubation was carried out for 48 h at 12.3 °C, 20 °C, 25 °C and 30 °C, respectively, using anaerobic conditions. Additionally, the growth and acidification behaviour of the two strains were assessed in MRS broth to confirm their previously reported very similar performance [30]. The initial cell concentration was set to 10² CFU/mL, and pH as well as viable anaerobic plate counts were assessed at several time points during incubation at 30 °C. pH was monitored using a pH meter (Model HI99161, Hanna Instruments, Temse, Belgium) and serial dilutions were plated on MRS agar plates using an Eddy Jet spiral plater (IUL instruments, Barcelona, Spain). Plates were incubated anaerobically at 30 °C and evaluated after 48 h. All experiments were conducted in triplicate, with each sample plated in duplicate.

### 2.4. Yoghurt Analogue Production

#### 2.4.1. Preparation of Starter Cultures

Prior to inoculation, frozen stock cultures of *L. sakei* 1.411 and 1.2307 were thawed and 100 µL was transferred into 9.9 mL of MRS broth, followed by incubation at 30 °C for 24 h. The activated cultures were subsequently washed to remove residual MRS broth by centrifugation at 650× *g* for 10 min at 20 °C using a Universal 320 R centrifuge (Hettich, Tuttlingen, Germany). The supernatant was discarded, and the resulting pellet was resuspended in 10 mL of filtered reverse osmosis (RO) water (Minisart, pore size 0.45 µm) and vortexed. This washing step was performed twice.

Prior to food matrix inoculation, the cultures were prepared as follows: In the first step, coconut milk enriched with either 2.5% *w*/*w* or 5.0% *w*/*w* sucrose was heated and stirred for 3 min at 90 °C using a Thermomix TM6 (Vorwerk, Wuppertal, Germany). After cooling, the coconut milk was inoculated with 0.01% *v*/*v* of the activated and washed cultures. The starter cultures were then incubated at the optimal growth temperature of 30 °C for 24 h prior to further use.

#### 2.4.2. Preparation of Yoghurt Analogues

**Figure 1:**
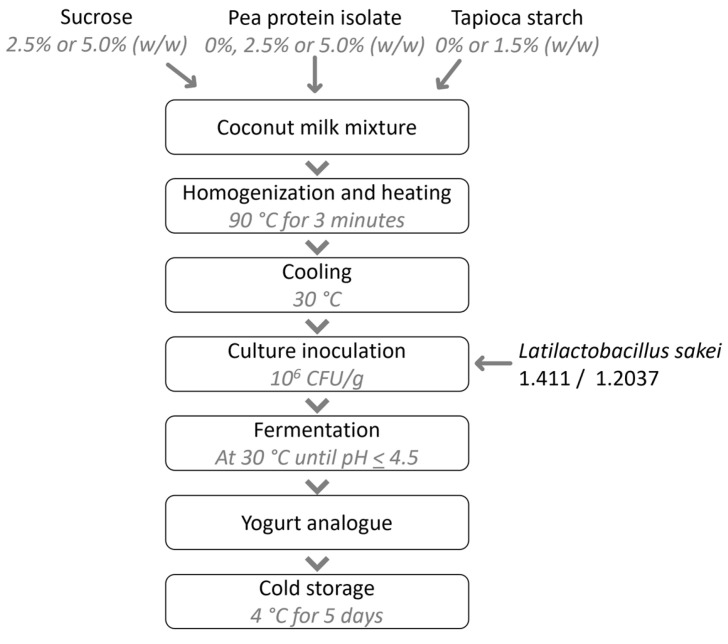
Flowchart of coconut yoghurt analogue production and storage.

The coconut-based yoghurt analogues were prepared following a modified protocol based on the method described by Pachekrepapol, et al. [45]. 950 mL of UHT coconut milk was mixed with sucrose (2.5 or 5.0% *w*/*w*), pea protein isolate (PPI; 0, 2.5 or 5.0% *w*/*w*), and/or tapioca starch (0 or 1.5% *w*/*w*). Samples without the addition of PPI and tapioca starch were also produced. The mixtures were stirred and heated to 90 °C for 3 min using a Thermomix TM6 at a speed of 2.5. The heated mixtures were transferred to 1000 mL beakers and cooled for 2 h in a water bath (30 °C) to reach a sample temperature of 30 °C, whereafter the prepared starter cultures (*L. sakei* 1.411 or 1.2307) were added at a concentration of ~10^6^ CFU/g. Test-dependent fermentation was conducted in different incubation tubes as further highlighted in the subsequent sections. The mixtures were incubated at 30 °C until a pH < 4.5 was reached, whereafter the yoghurt analogues were stored at 4 °C for 5 days. A flowchart of the process is presented in Figure 1.

#### 2.4.3. pH Changes During Fermentation and Cold Storage

The pH changes of the yoghurt analogues were measured using the HI996161 pH meter (Hanna Instruments, Woonsocket, RI, USA) at specific time points during fermentation (0, 2, 18, 20, 22, 24, 26 h) and on the first and fifth day of storage at 4 °C.

#### 2.4.4. (An)aerobic Viable Cell Counts

Viable cell counts were determined from samples collected at the start and end of fermentation, as well as on days 1 and 5 of storage at 4 °C. To monitor the starter cultures used, samples were serially diluted and plated in duplicate on MRS agar plates using the Eddy Jet spiral plater (IUL Instruments, Barcelona, Spain) and incubated anaerobically at 30 °C for 24–48 h. Additionally, samples from day 1 and 5 of storage were plated on PCA and incubated aerobically at 30 °C for 24–48 h. Afterwards, plates were counted, and in the case of PCA, also qualitatively assessed.

### 2.5. Influence of In Situ EPS Production on the Degree of Syneresis and Texture Development

#### 2.5.1. EPS Isolation and Quantification

A slightly modified method of Hilbig et al. [30] was used to isolate and quantify EPS present in the yoghurt analogues. Only yoghurt analogues formulated without starch were included in the analysis. Specifically, these comprised samples containing 2.5% or 5.0% *w*/*w* sucrose and either 2.5% or 5.0% *w*/*w* PPI, as well as control samples containing 5.0% *w*/*w* sucrose without added PPI or starch. Starch-containing formulations were excluded due to analytical interference. Samples were taken at the start and end of fermentation and kept frozen until further analysis. To isolate the EPS, samples were thawed and homogenized using a vortex mixer, followed by suspending 10 g in 20 mL of ethanol and incubating for 16 h at 4 °C. After incubation, samples were centrifuged at 6000× *g* for 15 min at 4 °C using the Universal 320 R Centrifuge (Hettich, Tuttlingen, Germany). The pellet, which contains the EPS, was then suspended in 5 mL of distilled water and dissolved at room temperature (<50 °C). Subsequently, 15 mL of trichloroacetic acid (26.6% *w*/*v*) was added to reach a final concentration of 20% *w*/*v*, and the mixture was stored on ice for 1 h to induce protein precipitation. To remove the precipitated proteins from the supernatant, the mixture was centrifuged at 13,000× *g* for 20 min at 4 °C. The supernatant was mixed with 2 vol of ethanol and stored overnight at 4 °C to precipitate the EPS. In the next step, the sample was centrifuged at 10,000× *g* for 10 min at 4 °C, and the resulting pellet was then dissolved in 6.5 mL distilled water and 0.5 mL perchloric acid (70%) to reach a final concentration of 5% *v*/*v*. EPS hydrolysis was carried out by incubating the samples in a water bath at 95 °C for 6 h. Subsequently, precipitated proteins were removed by centrifugation at 13,000× *g* for 10 min at 4 °C. The supernatant was then treated by shaking it for 3 min with a 1:1 mixture of a weak anionic (Dowex 66 free base) and a strong cationic ion exchanger (Dowe50WX4 hydrogen form) to eliminate salts and other ions from the solution. The ion exchanger was removed by centrifugation at 3000× *g* for 5 min at 4 °C. Finally, the obtained solutions were filtered using sterile CHROMAFIL^®^ syringe adaptor filters (pore size 0.45 μm; Ø 25mm; Macherey-Nagel GmbH & Co. KG, Duren, Germany) and filled into HPLC vials. The solutions were analyzed by HPLC using a Rezex RHM column (Rezex RHM, Phenomenex, Aschaffenburg, Germany) with a flow rate of 0.6 mL/min (ddH_2_O) at 75 °C, and an injection volume of 20 µL. The monosaccharides were detected with a refractive index (RI) detector at 40 °C and the results were compared to glucose and fructose standard curves.

#### 2.5.2. Determining Degree of Syneresis

The degree of syneresis of yoghurt analogues was assessed on day 1 and 5 of storage at 4 °C. The samples were fermented in 50 mL conical centrifuge tubes (according to the conditions described in Section 2.4.2) and centrifugated at 650× *g* for 10 min at 20 °C using the Universal 320 R centrifuge (Hettich, Tuttlingen, Germany). Syneresis was determined by the weight percentage of the supernatant after centrifugation and calculated as follows:

$$Syneresis\left(\%\right)=\frac{Weight\;of\;supernatant\;(g)}{Weight\;of\;product\;(g)}\times 100$$

Measurements were performed in triplicate.

#### 2.5.3. Texture Analysis

A backward extrusion test was conducted to evaluate the texture of the yoghurt analogues after 1 and 5 days of storage at 4 °C. Measurements were performed using a TAPlus texture analyzer (Lloyd Instruments Ltd., Bognor Regis, UK) and the software Nexygen Plus 4.0. Samples were filled into cylindrical containers (33 mm diameter, 70 mm height) to a height of 30 mm and fermented under the conditions described in Section 2.4.2. Texture was evaluated based on firmness (N), defined as the maximum positive force recorded during the test, derived from the time/force curve. The test was carried out under the following conditions: 10 N load cell; cylindrical probe (20 mm diameter, 100 mm length)—preload speed: 1 mm/s and preload force: 0.04 N; extension rate (test speed): 1 mm/s; and a compression depth of 25 mm into the sample surface. Measurements were performed in triplicate.

### 2.6. (Descriptive) Sensory Evaluation

A (descriptive) sensory evaluation was performed in-house with 18 trained participants on yoghurt analogue samples formulated with 5.0% *w*/*w* sucrose and varying levels of PPI (2.5 and 5.0% *w*/*w*) and tapioca starch (0 and 1.5% *w*/*w*). Sensory attributes (sweetness, acidity and off-odour) were assessed by smelling the samples, while textural attributes (smoothness and thickness) were evaluated by touching the samples. A 10-point scale was used for scoring the different products. Sensory attributes were rated from 0 (not perceptible) to 10 (intense) and texture attributes were rated from 0 (grainy or liquid/non-viscous) to 10 (smooth and thick/viscous). Participants also rated the overall acceptability of each sample, with 10 indicating “very likeable”.

### 2.7. Statistical Analysis

All yoghurt analogue matrices were produced in duplicate and acidification (pH), syneresis, texture analysis, and viable cell counts were always assessed in triplicate. EPS content was analyzed from all matrices (according to Section 2.5.1), with each measurement performed using additional technical duplicates. Means and standard deviation were calculated using Excel (Microsoft, Redmond, WA, USA). The software SPSS (IMB SPSS Statistics version 29.0.2.0 (20)) was used for all statistical tests.

The normal distribution of the sample sets was evaluated using the Shapiro–Wilk test and the Levene’s test was performed to look into the equality of the variances. A one-way ANOVA with Tukey’s post hoc test was performed on sample sets which had a normal distribution and equal variances. In cases where the variances were not equal, the Welch’s one-way ANOVA with the Games–Howell post hoc test was performed. Additionally, if normality was not met, the Kruskal–Wallis test was applied (non-parametric alternative to ANOVA). An independent samples *t*-test was conducted to assess whether there was a statistically significant difference between two independent groups of samples (comparison of the two strains or between two time points). In all statistical tests, a significance level (α) of 0.05 was used.

## 3. Results and Discussion

### 3.1. Initial Strain Characterization

The performance of starter cultures is highly influenced by the composition of the food matrix and the parameters used during processing. For instance, temperature not only affects growth rate and acidification kinetics but also the formation of EPS [46]. The temperature that promotes EPS production is thereby strain-dependent and EPS formation is also influenced by other stress-inducing factors. Hilbig, Loeffler et al. [47] explored the potential of *L. sakei* 1.411 for use in reconstructed ham, aiming to enhance its water-binding capacity and, consequently, the juiciness of the final product. However, the processing conditions applied (high salt concentrations and very low tumbling temperatures) were too harsh for the strain to produce sufficient EPS to achieve these effects. In contrast, a different outcome was observed in spreadable, raw fermented sausages, where substantial HoPS formation occurred during the exponential growth phase at 25 °C, allowing, e.g., for a significant fat reduction in Teewurst [30]. These findings highlight the critical role of fermentation conditions, which can either promote or hinder acidification and/or EPS production during the development of yoghurt analogues. These relationships were also highlighted by other authors who investigated the influence of temperature and other processing conditions on the production of HoPS such as dextran [48,49]. Screening of both *L. sakei* strains revealed that both strains exhibited very similar growth and acidification behaviour (Table 1) at 30 °C, with cell counts increasing from ~2 × 10^2^ CFU/g to 10^8^ CFU/g within 24 h of incubation, following a very similar growth pattern. This observation has been previously reported in the pre-seeding studies of Hilbig et al. [30,31].

**Figure A1:**
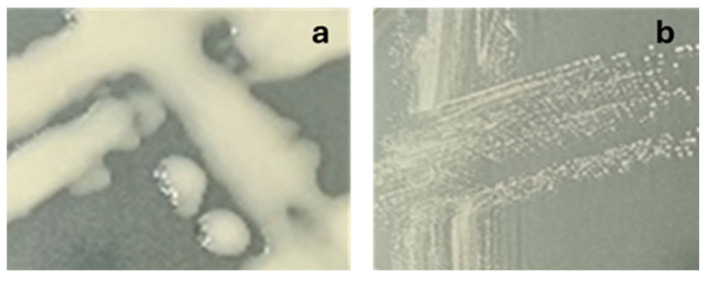
Modified MRS-agar with sucrose as the carbohydrate source incubated at 30 °C for 24 h with (**a**) *L. sakei* 1.411 (HoPS-producing strain), (**b**) *L. sakei* 1.2037 (non-EPS-producing strain).

*L. sakei* 1.411 was found to form mucoid (non-ropy) colonies indicative of HoPS production, which was the most pronounced at a fermentation temperature of 30 °C (Table 2). *L. sakei* 1.2037 formed smooth, non-mucoid colonies, and was subsequently used as a non-EPS producing control strain as already demonstrated in previous studies on meat products [30,50]. An example illustrating the colony morphology of both strains on sucrose-enriched PCA after 24 h of fermentation at 30 °C is provided in Appendix A (Figure A1).

Based on the results of the initial characterization, a fermentation temperature of 30 °C was selected for the yoghurt analogue fermentation.

### 3.2. Yoghurt Analogues

The compositional matrix of dairy yoghurts differs markedly from that of the vegan alternatives introduced in this study. While the gelation mechanisms in dairy yoghurt have been extensively characterized [9,22], the structural dynamics of pea protein-enriched coconut yoghurt present additional complexity, particularly when starch and/or in situ-produced HoPS are also present in the matrix. The latter aspects will be further discussed in the context of syneresis and texture analysis. The yoghurt analogues were produced from coconut milk, an oil-in-water emulsion, that was pre-heated and homogenized together with the added sugar, PPI and/or starch (Figure 1). The manufactured alternatives, especially those produced with 5% *w*/*w* PPI, exhibit a comparable or higher protein content than products currently available on the market, where coconut-based yoghurts typically contain around ≤1% protein, while legume-enriched coconut yoghurts generally provide ≥5 g protein per (mainly) 150 g serving size [51]. Plant proteins generally exhibit amphiphilic properties that enable them to act as “emulsifiers” by adsorbing at oil–water interfaces [52]. The PPI used in this study was found to be fully denatured, as confirmed by in-house differential scanning calorimetry (DSC) measurements, which influences the proteins’ solubility and emulsifying as well as gelling capacity to some extent. Remaining insoluble protein particles may also contribute to emulsion stability via a Pickering stabilization effect [53,54], where solid particles irreversibly adsorb at the oil–water interface, forming a steric barrier that inhibits droplet coalescence. Given the heterogeneous nature of PPI particles, the Pickering effect is, however, very likely to be much less pronounced than in systems employing engineered protein particles with optimized size and surface charge characteristics [53]. By combining the processing steps, homogenization, heating, and subsequent acidification through fermentation, stable gels could be formed, which effectively immobilized water, as is further explored in the sections on yoghurt analogue analysis.

In the present study, the influence of fermentation with *L. sakei* 1.411 on the properties of the pea protein-enriched yoghurt analogues was investigated, with a particular focus on syneresis and texture. The results were compared to yoghurt analogues fermented with a non-EPS producing control strain to better assess the role and impact of the in situ formed HoPS. The experimental design also enables a comparative analysis of the effects of protein enrichment and/or starch addition, thereby highlighting the potential and limitations of the proposed fermentation approach using the HoPS-former *L. sakei* 1.411.

#### 3.2.1. pH and Viable Cell Count During Storage Time

The yoghurt analogues were incubated with an initial cell concentration of around 10^6^ CFU/g with either the non-EPS producing strain *L. sakei* 1.2037 or the HoPS-forming strain *L. sakei* 1.411. During fermentation, the microbial cell counts increased to ~10^7^–10^8^ CFU/g by the time the pH of the yoghurt analogues reached 4.5, which was between 18 and 22 h of incubation. Furthermore, viable cell counts remained high during cold storage and were found to be 10^8^–10^9^ CFU/g at day 1 and 5. The pH values of the yoghurt analogues at the start and after 18 h (when most of the samples reached a pH of ≤4.5) of fermentation at 30 °C, as well as the pH values of yoghurt analogues measured on day 1 and day 5 of storage at 4 °C are shown in Table 3. The addition of PPI resulted in increased pH values at the start of incubation. Yoghurt analogues prepared with 2.5% *w*/*w* added sugar had significantly (*p* < 0.05) higher pH values compared to those prepared with 5.0% *w*/*w* added sugar, regardless of the *L. sakei* strain used or the storage day investigated, which is in accordance with other studies [55]. In some products, slightly but still significantly lower pH values were recorded on day 5 of storage as compared to day 1. This finding is in agreement with that of Pachekrepapol et al. [45], who also focused on coconut-based yoghurt alternatives.

#### 3.2.2. EPS Quantification

The quantification of the in situ-produced EPS in yoghurt analogue matrices fermented with *L. sakei* 1.2037 did not yield detectable levels, confirming its usage as a negative control for EPS production. Table 4 shows the quantification of dextran (determined as glucose monomers) in yoghurt analogue matrices fermented with *L. sakei* 1.411. Yoghurt analogues containing 5.0% *w*/*w* added sugar contained HoPS levels ranging from 0.690 ± 0.054 (only sucrose) to 0.799 ± 0.156 (+ 2.5% PPI), and 0.869 ± 0.002 (+ 5% PPI) g/100 g, respectively, compared to those with 2.5% *w*/*w* added sugar, which yielded lower but still pronounced HoPS concentrations. A study on in situ EPS production in non-fat set yoghurt from skimmed cow’s milk (5.2 g protein, 0.5 g fat and 4.7 g lactose) using different starter cultures (*Streptococcus thermophilus* and *Lactobacillus delbrueckii* subsp. *bulgaricus* (1:1), *Levilactobacillus brevis* UCLM-Lb47, *Leuconostoc mesenteroides* subsp. *mesenteroides* 2F6-9, and *Leuconostoc mesenteroides* subsp. *mesenteroides* 6F6-12) reported EPS concentrations ranging from 71.3 mg/L to 4.94 g/L, with the highest value obtained for *L. brevis* UCLM-Lb47 [56]. Ruikar et al. [57] investigated the dextran production of two strains of *Leuconostoc mesenteroides* and reported yields of 1.06 g/100 mL and 3.05 g/100 mL when cultured in a sucrose (30 g/L) broth medium at 28 °C for 48 h. A direct comparison with values reported in the literature is, however, challenging, as in situ EPS production is highly dependent on factors such as the bacterial strain, the composition of the matrix, and specific growth conditions. The observed increase in EPS concentration at higher initial sugar concentration can be attributed to substrate availability and was also reported by other authors. For example, Kuntiya et al. [58] showed that EPS concentration increased with sucrose concentration when *Lactobacillus confuses* was cultured in coconut water. Similarly, Satriadi et al. [59] reported that the influence of the sucrose addition enhanced the EPS production by *Lactobacillus acidophilus* and *L. plantarum*-fermented banana peels. The strain *L. sakei* 1.411 used in this study has been shown to produce high-molecular-weight dextran (under various conditions) in the presence of sucrose [29,60]. Polysaccharides with higher MW have been associated with the formation of more stable and rigid gel networks, contributing to increased resistance to deformation in, e.g., fermented dairy systems [61] as subsequently discussed in more detail.

#### 3.2.3. Syneresis

**Figure 2:**
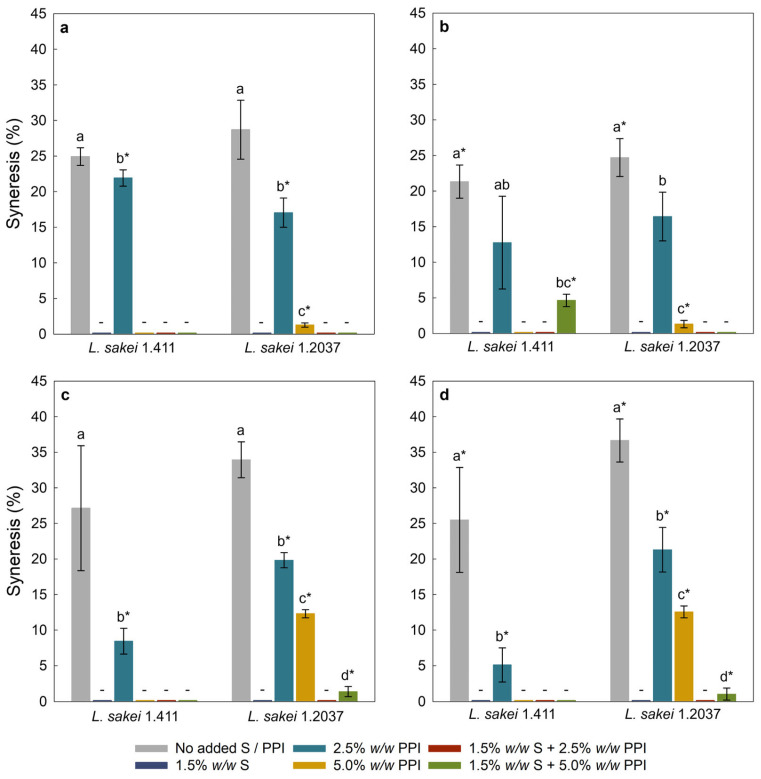
Syneresis of yoghurt analogues with 2.5% *w*/*w* sucrose at day 1 (**a**), 2.5% *w*/*w* sucrose at day 5 (**b**), 5.0% *w*/*w* sucrose at day 1 (**c**), and 5.0% *w*/*w* sucrose at day 5 (**d**) of storage at 4 °C. Within each graph, different letters indicate significant differences (*p* < 0.05) between yoghurt analogues fermented with the same *L. sakei* strain. An asterisk (*) indicates significant differences (*p* < 0.05) between identical yoghurt analogues fermented with different *L. sakei* strains. Error bars represent standard deviation (*n* = 3). ‘PPI’—pea protein isolate; ‘S’—tapioca starch; ‘-’—no syneresis.

Syneresis refers to the separation of liquid from the (vegan) yoghurt gel matrix [62]. It is a critical parameter in yoghurt manufacturing, as it reflects the yoghurt’s water-holding capacity during storage [63] and, hence, influences the perceived quality and texture of the final product. Figure 2 shows the syneresis of yoghurt analogues with 2.5% and 5.0% *w*/*w* added sucrose, with Figure 2a,c being the results from day 1, and Figure 2b,d being the results from day 5 of storage at 4 °C. Samples without starch and/or PPI addition showed the highest values of syneresis, regardless of the *L. sakei* strain used. Coconut milk is an oil-in-water emulsion containing less protein than cow’s milk, with protein contents of approximately 1.6% (as for the coconut milk used) and 3.5%, respectively. [64]. Therefore, it was not possible to form a cohesive gel network, which is essential for water retention and textural stability. Starch-containing samples showed no or very low syneresis, which is likely due to the strong gelling and water-binding properties of starch [65]. Lobato-Calleros et al. [66] and Ropciuc and Dabija [67] reported that yoghurt with added starch showed markedly lower syneresis than starch-free formulations. This effect is explained by the behaviour of gelatinized starch; upon heating, starch granules swell, absorb water and release amylose, thereby increasing the viscosity of the aqueous phase and improving water retention [68].

**Figure 3:**
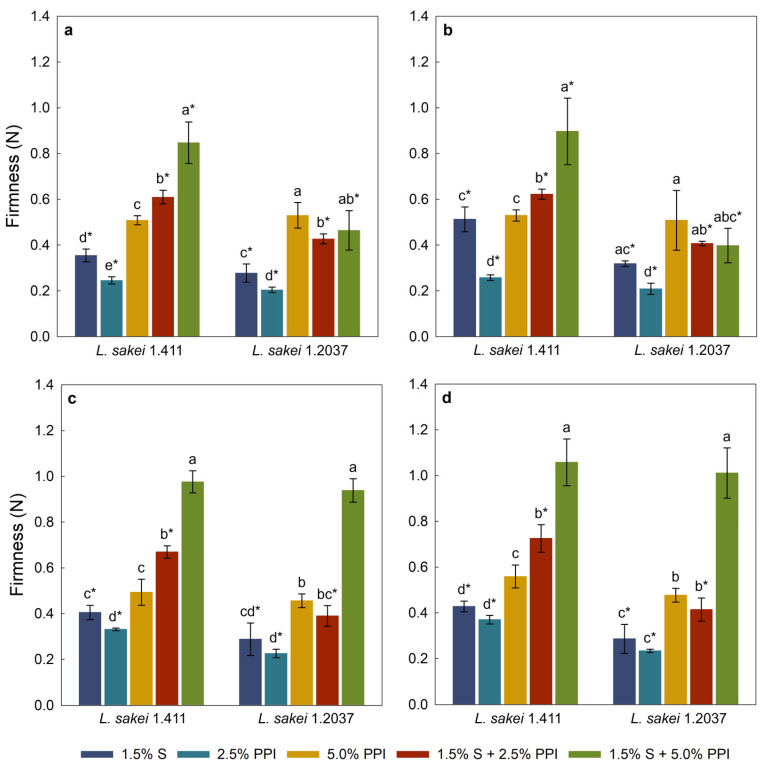
Firmness of yoghurt analogues with 2.5% *w*/*w* sucrose at day 1 (**a**), 2.5% *w*/*w* sucrose at day 5 (**b**), 5.0% *w*/*w* sucrose at day 1 (**c**), and 5.0% *w*/*w* sucrose at day 5 (**d**) of storage at 4 °C. Within each graph, different letters indicate significant differences (*p* < 0.05) between yoghurt analogues fermented with the same *L. sakei* strain. An asterisk (*) indicates significant differences (*p* < 0.05) between identical yoghurt analogues fermented with different *L. sakei* strains. Error bars represent standard deviation (*n* = 3). PPI—pea protein isolate; S—tapioca starch.

Most yoghurt analogues fermented with *L. sakei* 1.411 exhibited a slight decrease in syneresis over time, which can be ascribed to in situ formed EPS (*here* HoPS) that can bind and immobilize free water, thereby reducing syneresis and contributing to a denser, more compact gel network. This finding is supported by the texture analysis (Figure 3), where a slightly higher firmness was observed after five days of cold storage, indicating a denser and more compact gel structure. The findings are also in alignment with Pachekrepapol et al. [45], who observed a decrease in the syneresis of coconut-based yoghurt analogues with storage time (1–7–14 days). The *L. sakei* 1.411-fermented analogues formulated with added 2.5% *w*/*w* sucrose and 5.0% *w*/*w* PPI, in combination with starch, exhibited a significant increase in syneresis over time, which may occur due to the shrinkage or rearrangement of the gel network, reducing its ability to retain water [69]. Additionally, the lower added sucrose concentration (2.5% *w*/*w*) resulted in lower EPS concentrations as compared to samples containing 5.0% *w*/*w*, leaving less EPS available to bind (the released) water. Furthermore, samples containing 2.5% *w*/*w* PPI resulted in significantly lower syneresis when fermented with *L. sakei* 1.411 and supplemented with 5.0% *w*/*w* sucrose, compared to analogues fermented with the same strain but a lower sucrose concentration (2.5% *w*/*w*), as well as those fermented with *L. sakei* 1.2037 at both 2.5% and 5.0% *w*/*w* added sucrose, respectively. This reduction may be attributed to protein–polysaccharide interactions, enhanced by a higher EPS concentration in yoghurt analogues with 5.0% *w*/*w* added sucrose (Table 4), and water-holding ability as also shown in other studies [70]. An increased PPI concentration resulted in no syneresis, and significantly reduced syneresis for *L. sakei* 1.2037-fermented samples. The binding characteristics between EPS and proteins are strongly influenced by factors such as charge, molecular weight, protein surface exposure, and environmental conditions (e.g., ionic strength of the surrounding matrix). These interactions can be hydrophilic, hydrophobic, or electrostatic in nature, with electrostatic binding occurring between negatively charged EPS and positively charged proteins in a pH-dependent manner [71]. Such associative complexes have been shown to modify aggregation kinetics and, in some cases, delay gel formation, as observed in dairy yoghurts [72]. Additionally, weak hydrophobic interactions, hydrogen bonding, and physical entanglement contribute to bridging and network stabilization, which can reduce syneresis and influence perceived viscosity. Both in situ EPS production and polymer molecular characteristics determine whether EPS integrate into the forming gel or remain in the continuous phase, acting primarily as a thickener [73]. For neutral EPS, hydrogen bonding and physical entanglement are typically the dominant mechanisms. High-molecular-weight dextran can thereby act as a network-bridging agent by spanning protein clusters, thereby increasing mesh size and water retention, which slows gel network rearrangements and reduces syneresis [74]. A filler-like function has been reported in a study on the influence of added dextran on the (acid-induced) gel properties of faba bean protein isolates [75]. As previously mentioned, the strain *L. sakei* 1.411 has been found to produce high-molecular-weight dextran (under various conditions) in the presence of sucrose [29,60], supporting the results observed in the present study. One should, however, keep in mind that EPS molecular weights can also differ to some extent for the same strain, depending on the matrix and processing conditions applied [76]. In addition to protein-enriched yoghurt analogues, we produced variants with added tapioca starch, which affected both syneresis (Figure 2) and firmness (Figure 3). As discussed above, microstructural studies have shown that EPS can become part of the gel backbone by bridging protein clusters and influencing the mesh size of the formed gels, or they remain in the continuous phase. In contrast, starch primarily acts as a bulk thickener, increasing viscosity through granule swelling (gelatinization) and by occupying pore space [77]. When EPS contribute to network formation by enhancing protein connectivity and starch provides bulk viscosity, a synergistic effect may hence be observed, with combined systems showing greater reduction in syneresis and improved textural characteristics. Conversely, when dextran remains in the continuous phase (without bridging) and starch is the dominant thickener, the effect is merely additive [78]. Moreover, depending on the EPS characteristics, concentration and localization relative to starch domains, (exo)polysaccharides can have a positive or negative influence on starch retrogradation [77], with high-molecular-weight dextran being produced in situ being far more likely to act as a true network former (also promoting co-localization with forming protein aggregates). It may, therefore, have a stronger influence in retarding retrogradation in the plant-based yoghurt matrices than low-molecular-weight or ex situ-applied dextran. In addition, both the processing conditions and the type of protein used also affect protein–starch interactions, which in turn influence starch retrogradation over time in the yoghurt analogues [77,79].

#### 3.2.4. Texture Analysis

The texture of viscous foods such as yoghurt or mayonnaise is often characterized using the back-extrusion method, as it provides an objective assessment of properties like firmness, consistency, and flow behaviour [80]. The firmness results of yoghurt analogues prepared with 2.5% *w*/*w* and 5.0% *w*/*w* added sucrose are shown in Figure 3a,c for day 1, and Figure 3b,d for day 5 of storage at 4 °C. Most of the yoghurt analogues fermented with *L. sakei* 1.411 were significantly (*p* < 0.05) firmer compared to their counterparts fermented with *L. sakei* 1.2037. A similar effect of in situ-produced EPS on the firmness of dairy yoghurt was found by Jolly et al. [81], which was related to EPS–protein interactions. In addition, analogues fermented with the EPS-producing strain with 5.0% *w*/*w* added sucrose generally showed a trend towards higher firmness values compared to samples containing lower sucrose concentrations. This effect could be attributed to the higher EPS yield observed in those samples (Table 4), which likely enhanced water-binding capacity and promoted protein–polysaccharide interactions, thereby contributing to enhanced gel firmness. Pachekrepapol et al. [45] reported an increase in the rheological properties of coconut yoghurt analogues with different tapioca starch concentrations over the 1st, 7th and 14th day of storage at 4 °C, indicating enhanced viscoelastic characteristics over time. In the present study, firmness was only measured after 1 and 5 days of storage at 4 °C, which may not be sufficient to fully observe time-dependent changes. In general, samples containing starch and 5.0% *w*/*w* PPI had the highest firmness. The initial heating step caused starch granules to swell and gelatinize, which increased the system viscosity and promoted interactions with the protein network (e.g., by filling pores) and enhanced water binding, factors that collectively contribute to the structural integrity and stability of the yoghurt analogues. Comparing the firmness of yoghurt analogues containing 2.5% *w*/*w* and 5.0% *w*/*w* PPI reveals significant (*p* < 0.05) lower values in samples with the low PPI concentration. This suggests that higher PPI levels contribute to a denser and more cohesive gel network, likely due to increased protein–protein interactions and network formation during acid-promoted gelation [82]. All analogues, with the exception of the one made with 2.5% *w*/*w* sucrose and 5.0% *w*/*w* PPI, indicate the positive effect of starch combined with PPI on firmness, where a combination led to an increased firmness. This could be linked to the aforementioned ingredient interactions as also described by Velde et al. [83].

#### 3.2.5. (Descriptive) Sensory Evaluation

**Figure 4:**
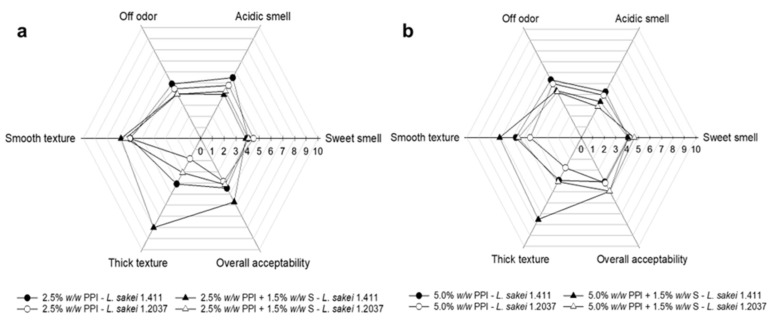
Sensory evaluation of yoghurt analogues prepared with 5.0% *w*/*w* sucrose containing (**a**) 2.5% *w*/*w* PPI or in combination with 1.5% *w*/*w* tapioca starch; (**b**) 5.0% *w*/*w* PPI or in combination with 1.5% *w*/*w* tapioca starch. All parameters were e evaluated on a 10-point scale (*n* = 18). PPI—pea protein isolate; S—tapioca starch.

In the present study, all yoghurt analogues underwent a general quality control, with particular attention also given to spore-forming bacteria. Although only samples generally considered microbially stable were used in the experiments, we opted for a sensory evaluation without tasting, as the full hurdle concept for this type of product should first be further strengthened. This decision was made to better understand EPS formation and its effects, while deliberately omitting the use of additional cultures (e.g., bacteriocin-producing strains) and preservatives that may impact gel formation and/or EPS formation. These parameters will be addressed in more detail in future studies (next level of complexity). Since samples with 5% *w*/*w* added sucrose showed more promising results with regard to EPS formation, syneresis and texture measurements; those samples were further analyzed in the sensory test. The sensory evaluation aimed to characterize the yoghurt analogues regarding sample odour and overall acceptability, as well as sample thickness and smoothness, which are parameters that can be assessed in relation to texture. The results are illustrated in Figure 4. Overall, *L. sakei* 1.411-fermented yoghurt analogues containing PPI (2.5 or 5.0% *w*/*w*) and in combination with tapioca starch scored higher on sample thickness and slightly higher in smoothness compared to their counterparts fermented with *L. sakei* 1.2037. A similar conclusion was obtained when analyzing firmness by backwards extrusion measurements (Section 3.2.4). A positive influence of in situ-formed EPS on texture-related attributes was also reported by Folkenberg et al. [84], who observed that dairy yoghurts fermented with EPS-producing strains resulted in higher sensory scores for mouthfeel thickness and creaminess. Moreover, there seemed to be a synergistic textural effect between starch and the in situ-produced HoPS being present in the matrix, as indicated by the remarkably higher scores given for texture (thickness). This should be further investigated in future sensory trials that also include mouthfeel evaluation.

Acidic smell was slightly less pronounced in samples containing 5.0% *w*/*w* PPI compared to 2.5% *w*/*w* PPI, which could be related to the higher PPI concentration masking the acid odour. Comparable scores for off-odour, sweet smell and overall acceptability were obtained for all samples.

## 4. Conclusions

This study demonstrates that both matrix composition and bacterial strain significantly influence the properties of coconut-based yoghurt analogues. A higher added sucrose concentration (5.0% *w*/*w*) led to increased EPS production by the HoPS-producing strain *L. sakei* 1.411, which contributed to improved water-holding capacity and enhanced gel properties. As a result, yoghurt analogues fermented with the EPS-producing strain, whether containing PPI alone or in combination with tapioca starch, showed lower syneresis and higher firmness compared to those fermented with the EPS-negative control strain. Overall, it was possible to produce stable, pea protein-enriched, coconut-based yoghurt analogues, with starch and/or EPS further enhancing the gel network. It should, however, be noted that EPS production alone did not achieve the same level of gel firmness as formulations that also included starch. While this may be advantageous for lower-viscosity yoghurt alternatives or plant-based yoghurt drinks, it also highlights that despite pronounced HoPS production, in situ-formed EPS alone may not fully replace the functional role of added hydrocolloids/thickeners such as starch. Overall, the results of this study underscore the potential of mesophilic HoPS-producing LAB to enhance the textural properties of yoghurt analogues, and future studies should aim to connect these results with further sensory aspects such as mouthfeel and taste perception. Further research could explore co-fermentation strategies aimed at reducing fermentation/acidification time while maintaining or even further enhancing EPS yields and product quality. To find a good balance between these factors will be of critical importance. Additionally, screening other EPS-producing strains, particularly HoPS producers, may offer opportunities to achieve higher EPS yields. These should then be examined in terms of sample microstructure, including interactions with components such as proteins and other hydrocolloids, using advanced microscopy techniques, to generate a deeper understanding that could facilitate product development and process optimization for large-scale manufacture. The incorporation of bacteriocin-producing strains during production could further contribute to the multi-hurdle approach, enhancing the microbial safety and shelf-life of plant-based yoghurt alternatives. Research in these directions would pave the way for potential industrial applications. However, product standardization must be ensured, which requires process optimization on a large scale. Furthermore, scalability and cost-effectiveness, considering factors such as production time and the use or reduction of hydrocolloids, should be evaluated under industrial conditions.

## Figures and Tables

**Table 1:** pH values at specific time points of fermentation at 30 °C with *L. sakei* 1.411 and *L. sakei* 1.2037 in MRS broth (*n* = 3).

		0 h	18 h	24 h
*L. sakei* 1.411	pH (-)	5.85 ± 0.08	5.29 ± 0.04	4.46 ± 0.08
*L. sakei* 1.2037	pH (-)	5.84 ± 0.06	5.14 ± 0.05	4.33 ± 0.03

**Table 2:** Growth and EPS formation of screened *Latilactobacillus sakei* strains with assessed EPS amounts based on mucoid colony appearance on MRS agar. (+++) strong, (++) medium, (+) weak (−) absent; (*n* = 3).

	Temperature (°C)	Growth	EPS Formation
*L. sakei* 1.411	12.3	−	−
	20.0	+	+
	25.0	++	++
	30.0	+++	+++
*L. sakei* 1.2037	12.3	−	−
	20.0	+	−
	25.0	++	−
	30.0	+++	−

**Table 3:** pH values of the yoghurt analogues measured at the start and after 18 h of incubation at 30 °C and after cold storage (*n* = 3). ‘PPI’—pea protein isolate; ‘S’—tapioca starch. An asterisk (*) indicates significant differences between day 1 and 5 of cold storage within the line.

			pH (-)			
	Added Sugar	Recipe	Start of Incubation	18 h of Incubation	Day 1	Day 5
*L. sakei* 1.411	2.5% *w*/*w*	No added S/PPI	6.03 ± 0.02	4.57 ± 0.02	4.46 ± 0.03 *	4.36 ± 0.01 *
		1.5% *w*/*w* S	6.10 ± 0.02	4.51 ± 0.01	4.56 ± 0.02 *	4.44 ± 0.01 *
		2.5% *w*/*w* PPI	6.35 ± 0.02	4.16 ± 0.03	4.50 ± 0.15	4.46 ± 0.11
		5.0% *w*/*w* PPI	6.45 ± 0.03	4.40 ± 0.03	4.63 ± 0.14	4.58 ± 0.04
		1.5% *w*/*w* S + 2.5% *w*/*w* PPI	6.48 ± 0.03	4.29 ± 0.02	4.63 ± 0.09	4.57 ± 0.07
		1.5% *w*/*w* S + 5.0% *w*/*w* PPI	6.40 ± 0.03	4.37 ± 0.01	4.60 ± 0.10	4.56 ± 0.10
	5.0% *w*/*w*	No added S/PPI	6.13 ± 0.03	4.36 ± 0.03	4.24 ± 0.12	4.15 ± 0.11
		1.5% *w*/*w* S	6.14 ± 0.06	4.52 ± 0.12	4.29 ± 0.10 *	4.18 ± 0.06 *
		2.5% *w*/*w* PPI	6.31 ± 0.01	4.03 ± 0.03	3.87 ± 0.08	4.03 ± 0.13
		5.0% *w*/*w* PPI	6.46 ± 0.02	4.25 ± 0.03	4.12 ± 0.07	4.13 ± 0.10
		1.5% *w*/*w* S + 2.5% *w*/*w* PPI	6.33 ± 0.08	4.20 ± 0.03	4.19 ± 0.09 *	4.03 ± 0.06 *
		1.5% *w*/*w* S + 5.0% *w*/*w* PPI	6.44 ± 0.07	4.24 ± 0.06	4.11 ± 0.11	4.07 ± 0.05
*L. sakei* 1.2037	2.5% *w*/*w*	No added S / PPI	6.04 ± 0.03	4.61 ± 0.01	4.45 ± 0.03 *	4.32 ± 0.03 *
		1.5% *w*/*w* S	6.10 ± 0.03	4.50 ± 0.02	4.44 ± 0.03 *	4.30 ± 0.03 *
		2.5% *w*/*w* PPI	6.28 ± 0.03	4.24 ± 0.03	4.46 ± 0.01 *	4.40 ± 0.03 *
		5.0% *w*/*w* PPI	6.44 ± 0.02	4.39 ± 0.04	4.42 ± 0.07	4.35 ± 0.06
		1.5% *w*/*w* S + 2.5% *w*/*w* PPI	6.38 ± 0.06	4.24 ± 0.02	4.46 ± 0.03 *	4.40 ± 0.02 *
		1.5% *w*/*w* S + 5.0% *w*/*w* PPI	6.39 ± 0.04	4.34 ± 0.02	4.43 ± 0.04	4.39 ± 0.03
	5.0% *w*/*w*	No added S/PPI	6.13 ± 0.03	4.47 ± 0.10	4.38 ± 0.06	4.19 ± 0.16
		1.5% *w*/*w* S	6.16 ± 0.04	4.51 ± 0.04	4.39 ± 0.06	4.35 ± 0.06
		2.5% *w*/*w* PPI	6.27 ± 0.04	4.28 ± 0.09	4.18 ± 0.05 *	4.20 ± 0.07 *
		5.0% *w*/*w* PPI	6.49 ± 0.04	4.28 ± 0.05	4.15 ± 0.07	4.23 ± 0.12
		1.5% *w*/*w* S + 2.5% *w*/*w* PPI	6.32 ± 0.04	4.23 ± 0.08	4.18 ± 0.09	4.17 ± 0.12
		1.5% *w*/*w* S + 5.0% *w*/*w* PPI	6.45 ± 0.02	4.25 ± 0.04	4.16 ± 0.04	4.15 ± 0.08

**Table 4:** Quantification of the in situ-produced HoPS (*n* = 4; determined as glucose monomers) of yoghurt analogues fermented with *L. sakei* 1.411. Yoghurt analogues fermented with *L. sakei* 1.2037 are excluded from the table due to the absence of detectable in situ EPS production. Different uppercase letters indicate significant differences between recipes at the same sugar concentrations. Different lowercase letters indicate significant differences between sugar concentrations within the same recipe.

Added Sugar Concentration	Recipe	EPS Concentration (g/100 g Yoghurt Analogue)
2.5% *w*/*w*	2.5% *w*/*w* PPI	0.427 ± 0.134 ^Aa^
	5.0% *w*/*w* PPI	0.426 ± 0.228 ^Aa^
5.0% *w*/*w*	No added S/PPI	0.690 ± 0.054 ^A^
	2.5% *w*/*w* PPI	0.799 ± 0.156 ^Ab^
	5.0% *w*/*w* PPI	0.869 ± 0.002 ^Ab^

## Data Availability

The datasets used and analyzed during the current study are available from the corresponding author on reasonable request.

## Abbreviations

The following abbreviations are used in this manuscript:

EPS	Exopolysaccharides
HoPS	Homopolysaccharides
LAB	Lactic acid bacteria
